# Protective Effects of Oridonin on Acute Liver Injury via Impeding Posttranslational Modifications of Interleukin-1 Receptor-Associated Kinase 4 (IRAK4) in the Toll-Like Receptor 4 (TLR4) Signaling Pathway

**DOI:** 10.1155/2019/7634761

**Published:** 2019-09-12

**Authors:** Min Shi, Yilin Deng, Heguo Yu, Ling Xu, Cuicui Shi, Jiong Chen, Guangming Li, Yiqi Du, Yu-gang Wang

**Affiliations:** ^1^Department of Gastroenterology, Changhai Hospital, Second Military Medical University/Naval Medical University, Shanghai 200433, China; ^2^Department of Gastrointestinal Cancer Biology, Tianjin Medical University Cancer Institute and Hospital, National Clinical Research Center for Cancer, Key Laboratory of Cancer Prevention and Therapy, Tianjin 300060, China; ^3^NPFPC Key Laboratory of Contraceptives and Devices, Shanghai Institute of Planned Parenthood Research (SIPPR), Institutes of Reproduction and Development, Fudan University, Shanghai 200000, China; ^4^Department of Gastroenterology, Shanghai Tongren Hospital, Shanghai Jiao Tong University School of Medicine, Shanghai 200336, China; ^5^Department of Gastroenterology, Xinhua Hospital, Shanghai Jiao Tong University School of Medicine, Shanghai 200092, China

## Abstract

**Objective:**

Recent researches have demonstrated that inflammation-related diseases are effectively regulated by posttranslational modifications (PTMs) including phosphorylation and acetylation. Our previous study found a new acetyltransferase inhibitor, oridonin, which had a protective effect on acute liver injury (ALI). In the present study, we further investigated its protective mechanism against D-galactosamine (D-Gal) combined with lipopolysaccharide- (LPS-) induced ALI in mice.

**Methods:**

Intraperitoneal injections of LPS (40 *μ*g/mouse)/D-Gal (5 mg/mouse) were given to the mice, and the experimental group was pretreated with intraperitoneal injection of oridonin (0.2 mg/mouse). To elucidate the protective mechanism of oridonin, we collected liver specimens and used RNA-sequencing (RNA-Seq) analysis. We focused on the genes that were upregulated by LPS/D-Gal and downregulated after pretreatment with oridonin. The downregulated genes examined by Gene Ontology (GO) and Kyoto Encyclopedia of Genes and Genomes (KEGG) pathway analysis were further verified by real-time polymerase chain reaction (PCR) and western blot.

**Results:**

GO analysis showed that genes that were downregulated after pretreatment with oridonin were extremely concentrated in immune response, chemotaxis, and inflammatory response. Real-time PCR confirmed that the expression of these genes was upregulated by LPS/D-Gal induction and reduced after treatment with oridonin, which was consistent with RNA-Seq results. KEGG pathway analysis showed a significantly enriched downregulated gene that was present in the Toll-like receptor (TLR) 4 signaling cascade. Our results manifested that phosphorylation levels of upstream signaling molecules in the TLR4 signaling cascade, including extracellular signal-regulated kinase (ERK), P38, and I*κ*B, were significantly inhibited by oridonin. Furthermore, LPS/D-Gal stimulation triggered posttranslational modifications of related gene loci in the TLR4 signaling pathway, including phosphorylation of IL-1 receptor-associated kinase 4 (IRAK4 T345/S346) and acetylation of IRAK4 (K34). However, after treatment with oridonin, the modification pattern of IRAK4 expression stimulated by LPS/D-Gal was suggestively attenuated.

**Conclusion:**

Our study revealed that the protective effects of oridonin on LPS/D-Gal-induced ALI mediated by inhibition of the PTMs of IRAK4, including phosphorylation of T345/S346 and acetylation of K34.

## 1. Introduction

Severe acute liver injury (ALI) is a rapid pathological process that can be life-threatening [1]. Hepatitis virus, adverse drug effects, alcohol abuse, metabolic syndrome, and other factors can lead to ALI. ALI is a common starting point in a series of liver diseases that can result in acute or chronic liver failure, cirrhosis, and hepatocellular carcinoma [2]. Because these liver diseases account for around 2 million deaths per year globally, the global burden of liver disease is anticipated to rise; therefore, therapies for ALI are urgently needed [3, 4].

More and more studies have found that posttranslational modifications (PTMs), for example, acetylation, methylation, phosphorylation, ubiquitination, and SUMOylation, can control protein activity, facilitate protein interactions, and change protein subcellular localization, leading to regulation of important signaling pathways [5–7]. PTMs have a critical role in the duration and extent of the inflammatory response in liver injury through targeting the signaling molecules [3, 8, 9]. Interpretation of the correlation between various protein modifications is, to some extent, one of the keys to elucidate the mechanisms of liver injury [4, 10]. Regulation of PTMs might act as a significant therapeutic target during the liver injury treatment [11, 12]. Furthermore, related efficient inhibitors are now being explored as novel directions in drug research [13, 14].

Oridonin is a well-known diterpenoid acquired from the Chinese medicinal herb *Rabdosia rubescens*, also recognized as Dong Ling Cao [15]. Over the last few decades, oridonin has gained widespread attention because of its complex role in cancer treatment, such as leukemia [16, 17], lymphogenous malignancy [18], hepatocellular carcinoma [19], and colorectal cancer [20]. However, it has been documented that oridonin has many other therapeutic effects, comprising anti-inflammation [21, 22], neuroprotection [23], and antihepatic fibrosis [24]. Our previous study demonstrated that oridonin had protective effects on ALI stimulated by LPS/D-Gal, manifested as an improved survival rate, ameliorated histological abnormalities, and impaired liver function [25]. Additionally, we discovered that oridonin acted as an inhibitor of several acetyltransferases, such as pCAF, P300, Tip60, and GCN5 [26]. Therefore, in the current study, we intended to demonstrate whether the underlying protection mechanism of oridonin in ALI was related to the regulation of PTMs.

## 2. Materials and Methods

### 2.1. Reagents

Oridonin (>98% purity; C_20_H_28_O_6_; M.W. 360.42), obtained from Selleck (USA), was suspended in dimethyl sulfoxide, stored at −20°C, and thawed prior to use. Lipopolysaccharide (LPS) (*Escherichia coli*, 0111:B4), D-Gal, and thiol-specific antioxidant (TSA) were purchased from Sigma (USA). A myeloperoxidase (MPO) assay kit was from Nanjing Jiancheng Bioengineering Institute (China). A ReverTra Ace qPCR RT Kit was purchased from Toyobo (Japan). A SYBR Green Real-time PCR Master Mix was acquired from ExCell Bio (China). RIPA lysis buffer was purchased from KeyGen Biotech (China). Antibodies against ERK, P-ERK, P38, P-P38, I*κ*B, P-I*κ*B, interleukin-1 receptor-associated kinase 4 (IRAK4), P-IRAK4 (T345/S346), histone 3 (H3), H4, *α*-tubulin, acetyl-*α*-tubulin (K40), and GAPDH were obtained from Cell Signaling Technology (USA). Pan-anti-acetyl-lysine monoclonal antibodies were generated by our own laboratory, as described previously [27]. Horseradish peroxidase- (HRP-) conjugated goat anti-mouse and goat anti-rabbit antibodies were from Beyotime (China).

### 2.2. Generation of Peptides and Anti-Acetyl-IRAK4 (K34) Monoclonal Antibodies

Synthesized peptide sequence of acetyl-IRAK4 was PQEGWKK (ac) LAVAIK. Peptides and anti-acetyl-lysine-IRAK4 (K34) monoclonal antibodies were manufactured by GL Biochem (Shanghai, China).

### 2.3. Cell Culture and Assay of Acetyltransferase Inhibitory Activity of Oridonin

The mouse immortalized stellate cell line JS1 was acquired from Cell Resource Center (Shanghai Institutes for Biological Sciences, China). These cells were grown in Dulbecco's modified Eagle's medium with 10% fetal bovine serum and 1% penicillin/streptomycin at a 37°C incubator with 5% CO_2_. JS1 cells were seeded in 10 cm dishes and incubated in culture medium for 24 h. The cells were pretreated with oridonin (5, 10, and 15 *μ*m) for 3 h before adding TSA (1 mM). The cells were harvested after 4 h of TSA treatment. We performed western blot to detect the level of H3, H4, acetylated histone H3, acetylated histone H4, *α*-tubulin, and acetylated *α*-tubulin and compared with GAPDH as a control.

### 2.4. Animals

Animals were handled as per the institutional animal care policies of the Shanghai Institute of Planned Parenthood Research. Female C57BL/6 mice (specific pathogen-free, 20-22 g, wild type) were obtained from SIPPR-BK Animal Co. Ltd. (Shanghai, China). The mice were given with a regular chow diet and water. They were accommodated under standard laboratory environments (21 ± 2°C, 12 h light/dark cycle) and were adapted for a minimum of 2 weeks before beginning any experiment.

### 2.5. Experimental Design

ALI was prompted in mice with intraperitoneal injection of LPS (40 *μ*g/0.5 mL) combined with D-Gal (5 mg/0.5 mL). Animals were randomly separated into five groups (*n* = 6 each): control group (a), ALI group (b), two oridonin treatment groups (c and d), and oridonin control group (e). In group c, oridonin (0.2 mg/0.5 mL) was given 1 h prior to LPS/D-Gal challenge, and in group d, oridonin (0.2 mg/0.5 mL) was given every 4 d for a total of three doses, where the final dose was given 1 h before LPS/D-Gal challenge. All animals were killed by amputation at 6 h following LPS/D-Gal challenge. The liver tissues were acquired and preserved at −80°C for future use.

### 2.6. Transcriptome Analysis of Gene Expression Profiles of ALI upon Oridonin Treatment and Validation of the Differentially Expressed Genes (DEGs) by Real-Time Quantitative Polymerase Chain Reaction (qPCR)

The total mRNAs of five groups were isolated and analyzed using next-generation RNA-sequencing (RNA-Seq) technology on an Illumina HiSeq 2000 platform (Genenergy Bio, Shanghai, China) to outline their global gene expression patterns. For qPCR verification, total RNA was collected from five groups with or without oridonin treatment. Fluorescent qPCR was carried out on an ABI 7500 Fast Real-Time PCR detective system (Applied Biosystems, CA, USA) for chemokines and inflammatory cytokines. The SYBR Green qPCR system was 20 *μ*L in total, which consisted of 10 mL SYBR Premix Ex Taq, 1.6 mL of the forward and reverse primers, 7 mL of double-distilled water, and 1.4 mL cDNA template. The reaction conditions were performed according to the supplier's instructions. GAPDH served as a control. All samples were run in triplicate. The relative expression was calculated by the 2^−*ΔΔ*Ct^ method.  Table 1 shows the list of primers.

### 2.7. Hepatic Myeloperoxidase (MPO) Activity Assay

Activity of MPO was measured via spectrophotometry using the MPO assay kit.

### 2.8. Protein Isolation and Immunoblot

RIPA lysis buffer with protease inhibitor cocktail was used to extract the total protein. Protein concentrations were measured via the BCA method. Equivalent quantities of protein were utilized to examine ERK, P-ERK, P38, P-P38, IRAK4, P-IRAK4 (S345/T346) and acetyl-IRAK4 (K34), I*κ*B, P-I*κ*B, H3, H4, *α*-tubulin, acetyl-*α*-tubulin (K40), and pan-acetylation in comparison with GAPDH as a control. Goat anti-rabbit or goat anti-mouse IgG–HRP antibody was the secondary antibody. An enhanced chemiluminescence (ECL) detection kit was utilized to visualize the protein bands.

### 2.9. Statistical Analysis

All results are showed as the mean ± SD and from a minimum of three different independent experiments. Student's *t*-test was utilized to determine the difference between two groups. A *χ*^2^ test was utilized to the evaluation of sample rates among multiple samples. All analyses were accomplished with GraphPad Prism 6. *P* < 0.05 and *P* < 0.01 signified statistical significance.

## 3. Results

### 3.1. Oridonin Decreased Acetylation of H3, H4, and *α*-Tubulin in JS1 Cells and LPS/D-Gal-Induced ALI

**Figure 1:**
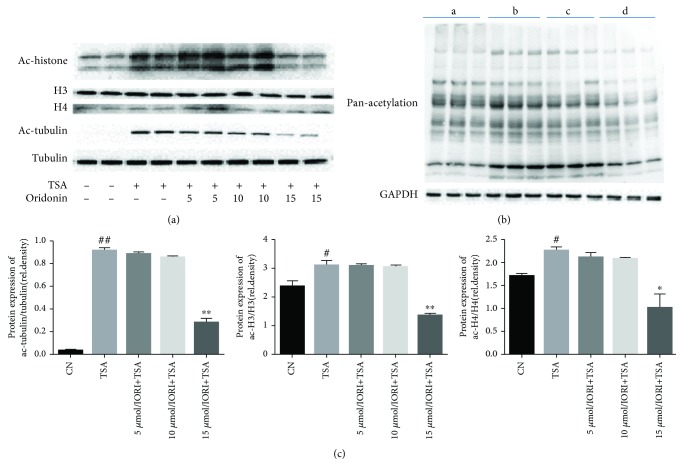
Acetyltransferase inhibitory activity of oridonin was assessed *in vivo* and *in vitro*. (a) JS1 cells were seeded in 10 cm dishes and incubated for 24 h. The cells were pretreated with oridonin (5, 10, or 15 *μ*m) for 3 h before adding 1 mM TSA. The cells were harvested after 4 h of TSA treatment. Western blot showed that oridonin decreased acetylation of H3, H4, and *α*-tubulin induced by TSA. The inhibitory effect of oridonin was obvious when the concentration was 15 *μ*m. (b) There was a substantial rise in the overall pan-acetylation level, especially expression of acetyl-H3, acetyl-H4, and acetyl-*α*-tubulin in LPS/D-Gal-induced ALI. The overall pan-acetylation level reduced in a concentration-dependent way upon oridonin treatment. (c) Band intensity in (a) was quantified by ImageJ. ^#^*P* < 0.05, ^##^*P* < 0.01 vs. control group; ^∗^*P* < 0.05, ^∗∗^*P* < 0.01 vs. TSA treatment group.

To assess the acetyltransferase inhibitory activity of oridonin *in vitro* and *in vivo*, we studied manifestation of acetyl-*α*-tubulin (K40), acetyl-H3, and acetyl-H4. The results indicated that the acetylation level of tubulin (K40), H3, and H4 decreased suggestively when the final concentration of oridonin was 15 *μ*m (Figure 1(a)). There was a noteworthy rise in the overall pan-acetylation level, especially the expression of acetyl-H3, acetyl-H4, and acetyl-*α*-tubulin in LPS/D-Gal-induced ALI. However, the overall pan-acetylation level reduced in a concentration-dependent way upon oridonin treatment (Figure 1(b)). Our study established that oridonin harbors the activity of acetyltransferase inhibitor *in vitro* and *in vivo*.

### 3.2. Bioinformatics Revealed the GOs and Signaling Pathways Regulated by Oridonin

**Figure 2:**
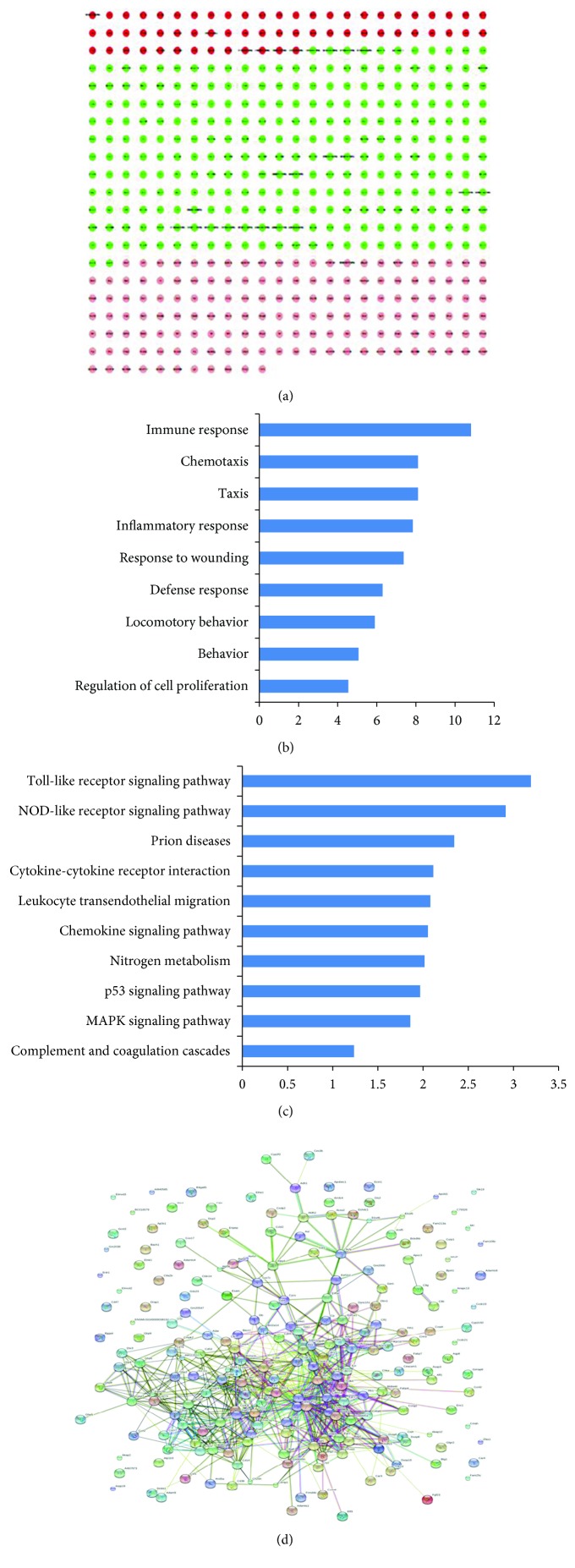
Effects of oridonin on gene expression profiles in LPS/D-Gal-induced ALI assayed by RNA-Seq. (a) Clustering analysis of gene expression profiles prompted by LPS/D-Gal challenge using Cytoscape software. The green part represents the genes that were upregulated by LPS/D-Gal. We focused on the target genes stimulated by LPS/D-Gal and downregulated by oridonin treatment in group d. The target genes were subjected to bioinformatics analysis. (b) GO analysis of target genes for biological processes revealed that downregulated genes were greatly enriched in immune response, chemotaxis, and inflammatory response. (c) KEGG pathway analysis of target genes showed that downregulated genes were enriched in several pathways including TLR and NOD-like receptor signaling pathways. (d) Interactions of the target genes demonstrated by KEGG pathway analysis.

We focused on the genes that were induced by LPS/D-Gal compared with the control group and then downregulated by oridonin treatment. Screening analysis indicated that LPS/D-Gal stimulated expression of 581 genes. In the oridonin-treated group c, 121 genes with fold changes ≥ 2 were downregulated by oridonin (Supplementary [Supplementary-material supplementary-material-1]). GO analysis suggested that the downregulated genes were extremely enriched in chemotaxis, locomotor activity, inflammatory response, and immune response. KEGG pathway analysis presented abundance of downregulated genes in several cascades, comprising the NOD-like receptor signaling pathway, mitogen-activated protein kinase (MAPK) signaling pathway, and TLR signaling pathway (Supplementary [Supplementary-material supplementary-material-1]). In the oridonin-treated group d, 278 genes with fold changes ≥ 2 were downregulated by three doses of oridonin (Supplementary [Supplementary-material supplementary-material-1]). GO analysis showed that the downregulated genes were greatly enriched in immune response, chemotaxis, and inflammatory response. KEGG pathway analysis indicated enhancement of downregulated genes in several cascades such as TLR and NOD-like receptor signaling pathways. Our previous research revealed that the prophylactic effects of oridonin were more obvious in group d, so we used the bioinformatics interpretation of group d, which better suggested the target of oridonin (Figure 2).

### 3.3. Significantly Enriched GO Term-Related Genes Verified by Real-Time PCR

**Figure 3:**
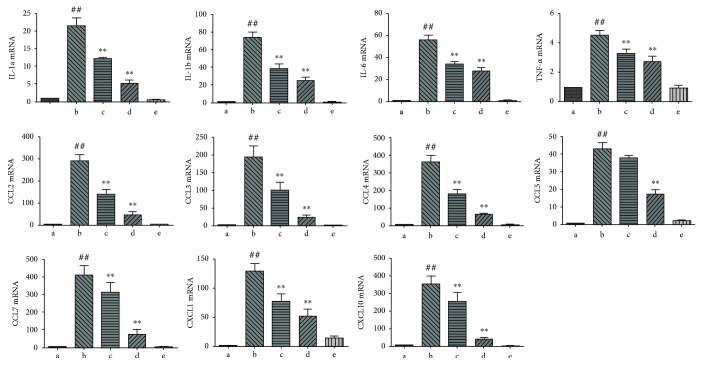
Significantly enriched GO term-related genes were validated by real-time PCR. Constant with RNA-Seq results, expression of GO term-related genes was elevated in the LPS/D-Gal group and decreased in a concentration-dependent way by oridonin treatment. ^##^*P* < 0.01 vs. control group (a); ^∗∗^*P* < 0.01 vs. model group (b).

To verify the outcomes of RNA-Seq, the genes involved in chemotaxis, immune, and inflammatory responses were subjected to real-time PCR. Hepatic expression of 11 chemotaxis and immune and inflammatory response-related genes (IL-1*α*, IL-1*β*, IL-6, TNF-*α*, CCL2, CCL3, CCL4, CCL5, CCL7, CXCL1, and CXCL10) was analyzed among the five groups. Coherent with RNA-Seq outcomes, expression of these 11 genes was elevated in the LPS/D-Gal group and reduced due to oridonin treatment. Oridonin suppressed chemotaxis, immune, and inflammatory response-related genes in a concentration-dependent way. These outcomes provide more evidence for the reliability of bioinformatics analysis, indicating that oridonin suppressed the production of cytokines involved in the immune and inflammatory processes, which might protect the liver from injury (Figure 3).

### 3.4. Oridonin Suppressed MPO Activity Prompted by LPS/D-Gal

**Figure 4:**
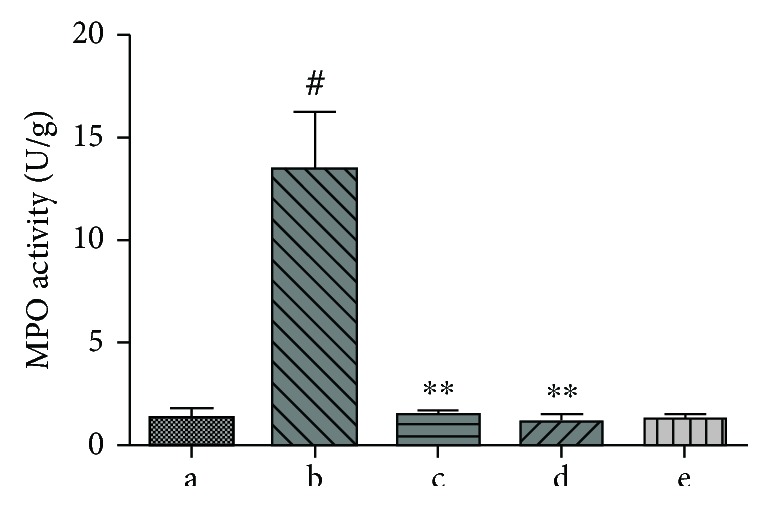
Oridonin suppressed MPO activity prompted by LPS/D-Gal. ^#^*P* < 0.05 vs. control group (a); ^∗∗^*P* < 0.01 vs. model group (b).

Inflammatory response, including excessive inflammation-related cell activation and infiltration into liver tissues, contributes to LPS/D-Gal-induced liver injury [28]. A large amount of neutrophil infiltration was observed in an ALI model, characterized by high levels of MPO activity. Pretreatment with oridonin inhibited neutrophil infiltration into liver tissues as demonstrated by reduced MPO activity (Figure 4).

### 3.5. Participation of NF-*κ*B and MAPK Signaling Pathways in the Anti-Inflammatory Impact of Oridonin on LPS/D-Gal-Induced ALI

**Figure 5:**
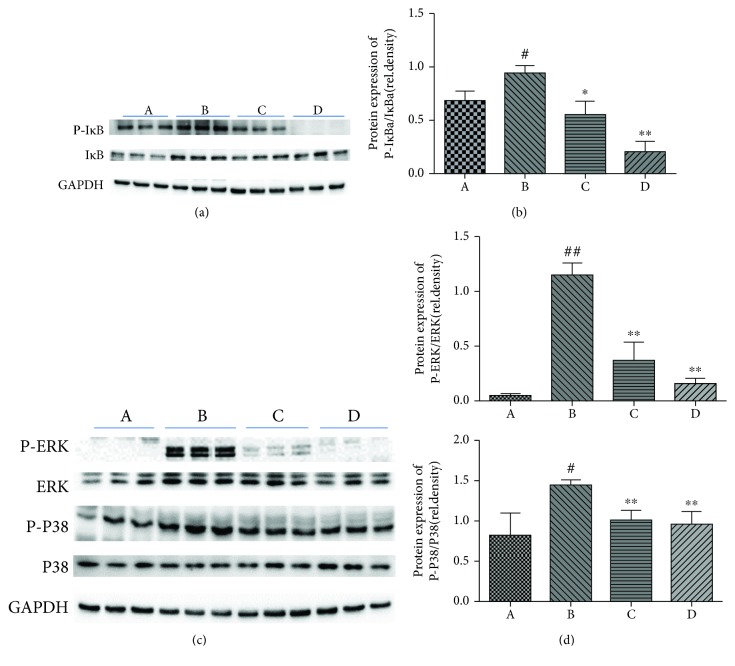
Participation of MAPK plus NF-*κ*B signaling pathways in the anti-inflammatory effect of oridonin on LPS/D-Gal-induced ALI. (a) Level of P-I*κ*B was inhibited by oridonin in a concentration-dependent way, which was prompted by LPS/D-Gal challenge (*P* < 0.05). (b) Band intensity in western blot was quantified by ImageJ. (c) Compared to the control group, the phosphorylation levels of MAPK (ERK as well as P38) were suggestively elevated in the ALI group (*P* < 0.01). Pretreatment with oridonin significantly inhibited phosphorylation of MAPK (*P* < 0.01). (d) Band intensity in western blot was computed using ImageJ. ^#^*P* < 0.05, ^##^*P* < 0.01 vs. control group (A); ^∗^*P* < 0.05, ^∗∗^*P* < 0.01 vs. model group (B).

KEGG analysis specified that the TLR4 signaling pathway was significantly enriched (Figure 2(c)). NF-*κ*B and MAPK are two critical downstream cascades participating in LPS-stimulated TLR4 signal transduction [29]. Stimulation of the MAPK pathway causes initiation of transcription factor AP-1, which plays a critical role in the regulation of proinflammatory cytokines [30]. We established that the elevated levels of P-ERK and P-P38 prompted by LPS/D-Gal were abolished by pretreatment of mice with oridonin. NF-*κ*B also has a vital role in controlling downstream signaling events that mediate proinflammatory gene transcription. In resting cells, NF-*κ*B is preserved in the cytoplasm by I*κ*B inhibitors, which are phosphorylated and then quickly degraded due to stimuli, resulting in NF-*κ*B nuclear activation [31]. It means that we could use the phosphorylation of I*κ*B to indicate the activation of NF-*κ*B. Thus, we analyzed the phosphorylation of I*κ*B to further elucidate the anti-inflammatory mechanism of oridonin. As expected, phosphorylation of I*κ*B was depressed by oridonin in a concentration-dependent way (Figure 5).

### 3.6. Modification (Phosphorylation and Acetylation) of IRAK4 Was Inhibited in LPS/D-Gal-Induced ALI by Oridonin

**Figure 6:**
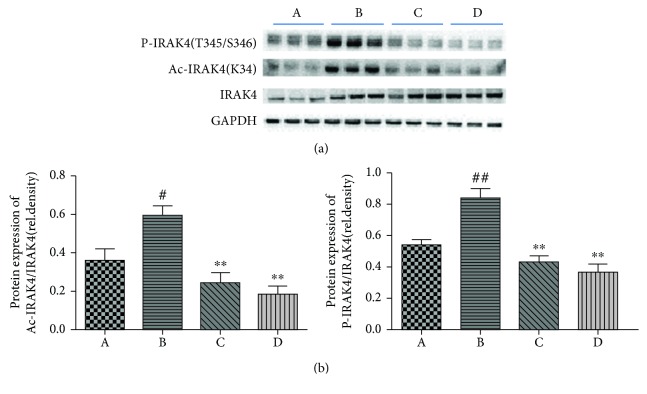
Modifications (phosphorylation and acetylation) of IRAK4 were impeded in LPS/D-Gal-induced ALI upon oridonin treatment. (a) Oridonin inhibited expression of P-IRAK4 (T345/S346) and acetyl-IRAK4 (K34) prompted by LPS/D-Gal. (b) Band intensity in western blot was quantified using ImageJ. ^#^*P* < 0.05, ^##^*P* < 0.01 vs. control group (A); ^∗∗^*P* < 0.01 vs. model group (B).

Protein PTMs have a deep impact on protein activity and stability along with binding capacity [32]. IRAK4 is indispensable for the MyD88-dependent TLR4 signaling pathway, including the activation of MAPK and NF-*κ*B signaling cascades [33]. Initial studies revealed that the kinase activity of IRAK4 is required for the proper activation of MAPK and NF-*κ*B [34]. Furthermore, another study using LC-MS/MS analysis recognized three phosphorylation sites (T342, T345, and S346) within the stimulation loop, which are critical to the kinase activity of IRAK4 [35]. Our results indicated that oridonin inhibited expression of P-IRAK4 (T345/S346) induced by LPS/D-Gal. IRAK4 is presumed to interact with MyD88 through the death domain, and the downstream signaling pathway is blocked without their interaction [36]. UniProt database indicated that the K34 site that resides in the death domain could be acetylated. The acetylation status of IRAK4 (K34) may influence the interaction between IRAK4 and MyD88, resulting in the blockade of signal transduction. Thus, we further identified that oridonin inhibited the acetylation level of IRAK4 (K34) stimulated by LPS/D-Gal. All the results suggested that LPS/D-Gal-induced PTMs of IRAK4 and oridonin pretreatment reversed the changes. The impediment of PTMs of IRAK4 may be one of the reasons of the anti-inflammatory mechanisms of oridonin (Figure 6).

## 4. Discussion

An established model for studying ALI in mice involves coadministration of LPS plus D-Gal, which results in a pathological progression parallel to clinical ALI and is also an imperative platform for screening novel medications for the treatment of ALI [37]. Our previous researches have shown that oridonin improves the survival rate of mice and liver histopathology and reduces plasma alanine and aspartate aminotransferase levels, indicating that oridonin has significant protective effects on ALI [25].

We further explored the possible mechanisms underlying oridonin-mediated hepatoprotection. Transcriptome analysis indicated that the protective mechanism of oridonin might be correlated with suppression of TLR4-mediated immune and inflammatory responses. The MPO activity of the liver, which is believed to be a key step in the initiation of the inflammatory response, was markedly reduced by oridonin treatment. The parallel inhibition of chemotaxis and immune as well as inflammatory response-related genes' mRNA expression implies that obstruction of transcription might have a crucial role in the protective impact of oridonin. The production of inflammatory mediators is known to depend mainly on activation of transcription factor NF-*κ*B [38]. The crucial step in NF-*κ*B stimulation is I*κ*B phosphorylation by IKK, and then, NF-*κ*B enters the nucleus and controls manifestation of inflammatory cytokines [38]. Activation of the MAPK family has a crucial function in a large number of cellular processes, including proliferation, differentiation, and expression of proinflammatory factors [39]. Our results revealed that oridonin inhibited the stimulation of NF-*κ*B. The inhibitory effect was demonstrated by the reduction of I*κ*B phosphorylation. At the same time, activation of the MAPK family members was inhibited by oridonin treatment. These results presented that the anti-inflammatory impact of oridonin is partly dependent on the MAPK and NF-*κ*B signaling pathways, which indicates that the target of oridonin might be stimulated earlier due to LPS/D-Gal challenge.

TLR4 signaling originally causes congregation of the LPS/TLR4 recognition complex, which prompts a cascade of PTMs of proteins, for example, IRAK4. The crucial involvement of IRAK4 in TLR4-mediated inflammatory responses has been documented in knockout (KO) studies [35]. Be in composition to IRAK1 KO mice, IRAK4 KO mice were absolutely unaffected to LPS-induced septic shock and did not have a cytokine response when challenged with numerous TLR ligands. Additionally, IL-1-stimulated NF-*κ*B, JNK, and p38 expressions were rigorously faulty in cells deficient in IRAK4. LPS-induced stimulation of JNK was also repressed, and LPS-induced NF-*κ*B stimulation was postponed [33]. Furthermore, some studies documented that the kinase activity of IRAK4 was vital for its action. Using IRAK4-deficient murine embryonic fibroblasts that were reconstructed with an IRAK4 kinase inactive mutant, researchers indicated that the kinase activity of IRAK4 was necessary for the best stimulation of IL-1-induced NF-*κ*B, JNK activation, and proinflammatory cytokines [34]. What is more, studies on IRAK4 kinase inactive knock-in mice showed that the kinase activity of IRAK4 is necessary for its action [40, 41]. One study reported there are three phosphorylation sites (T342, T345, and S346) that are responsible for IRAK4 kinase activity [35]. As expected, our results indicated that LPS/D-Gal-stimulated IRAK4 activation demonstrated by upregulation of P-IRAK4 (T345/S346), which was inhibited by oridonin treatment. However, macrophages from IRAK4 kinase inactive knock-in mice indicated that the kinase activity of IRAK4 was necessary for the stimulation of IL-1/TLR4-induced NF-*κ*B [40, 41]. Moreover, there was obvious diminishing of IL-1/TLR-induced cytokine as well as chemokine generation in IRAK4 kinase inactive knock-in mice [41–43]. Why are there two opposite conclusions? At present, different cell types are the causes of different results, and the specific mechanism has yet to be further studied. However, at least it is certain that IRAK4 kinase activity is necessary for the optimal inflammatory signaling pathway mediated by IL-1/TLR4.

All IRAK family members share analogous domain structures, comprising a conserved N-terminal death domain plus a central kinase domain [44]. IRAK4 binds to the adaptor protein MyD88 via its death domain. The downstream signaling pathway fails to activate without combination of the two proteins [45]. Thus, we infer that the death domain of IRAK4 has a crucial role in molecular recognition and activation of the downstream signaling pathway. Protein acetylation and the reverse process, named deacetylation, are PTMs. Histone acetyltransferases (HATs) as well as histone deacetylases (HDACs) could control gene expression by modifying histone proteins [46]. Nonetheless, HAT and HDAC can modulate precise signaling cascades and have additional targets apart from histones, comprising NF-*κ*B, signal transducer, and activator of transcription 3 (STAT3) as well as p53 [47–50]. Scientists have identified 3600 lysine acetylation sites on 1750 proteins, and the modification preferentially targets large macromolecular complexes [51]. Their findings support the concept that acetylation might control innate immune response-related proteins, for example, DDX58, IRAK4 (K34), OAS2, and TRIM25 [51]. K34, the acetylation modification target of IRAK4, is located in the death domain of IRAK4. Our results suggested that LPS/D-Gal can upregulate the level of acetyl-IRAK4 (K34), and we speculated that the acetylation of IRAK4 (K34) may promote the binding of IRAK4 to MyD88, thus enabling activation of downstream MyD88-dependent signaling pathways. Our results showed that oridonin pretreatment with LPS/D-Gal-induced ALI inhibits acetylation of IRAK4, resulting in blockade of the TLR4 signaling pathway.

However, our study had certain limitations, which require further studies. First of all, our results that could not provide direct and sufficient evidence to support the acetylation status of IRAK4 (K34) may influence the combination of MyD88 and IRAK4. We need to construct the wild type, site-modified type, and mutant type of IRAK4 to further clarify the different binding capacities with MyD88. Second, we should further identify whether these two PTMs are cross-linked.

## 5. Conclusions

In conclusion, our study revealed that the protective effects of oridonin on LPS/D-Gal-induced ALI are mediated by inhibition of the PTMs of IRAK4, including phosphorylation of T345/S346 and acetylation of K34.

## Figures and Tables

**Table 1:** Sequence of primers used for qPCR.

Target genes	Forward primers	Reverse primers
IL-1*α*	5′-AGT ATC AGC AAC GTC AAG CAA-3′	5′-TCC AGA TCA TGG GTT ATG GAC TG-3′
IL-1*β*	5′-GAA ATG CCA CCT TTT GAC AGT G-3′	5′-TGG ATG CTC TCA TCA GGA CAG-3′
IL-6	5′-TAG TCC TTC CTA CCC CAA TTT CC-3′	5′-TTG GTC CTT AGC CAC TCC TTC-3′
TNF-*α*	5′-CAG GCG GTG CCT ATG TCT C-3′	5′-CGA TCA CCC CGA AGT TCA GTA G-3′
CCL2	5′-TAA AAA CCT GGA TCG GAA CCA AA-3′	5′-GCA TTA GCT TCA GAT TTA CGG GT-3′
CCL3	5′-TGT ACC ATG ACA CTC TGC AAC-3′	5′-CAA CGA TGA ATT GGC GTG GAA-3′
CCL4	5′-TTC CTG CTG TTT CTC TTA CAC CT-3′	5′-CTG TCT GCC TCT TTT GGT CAG-3′
CCL5	5′-GTG CCC ACG TCA AGG AGT AT-3′	5′-GGG AAG CTA TAC AGG GTC A-3′
CCL7	5′-CCA CAT GCT GCT ATG TCA AGA-3′	5′-ACA CCG ACT ACT GGT GAT CCT-3′
CXCL1	5′-ACT GCA CCC AAA CCG AAG TC-3′	5′-TGG GGA CAC CTT TTA GCA TCT T-3′
CXCL10	5′-CCA AGT GCT GCC GTC ATT TTC-3′	5′-TCC CTA TGG CCC TCA TTC TCA-3′
GAPDH	5′-TCC AAG GAG TAA GAA ACC CTG GAC-3′	5′-GTT ATT ATG GGG GTC TGG GAT GG-3′

## Data Availability

The data used to support the findings of this study are available from the corresponding author upon request.

## Supplementary Materials

Supplementary Materials See Supplementary Tables S1-S2 in the Supplementary Materials for comprehensive image analysis. Figure S1: A. GO analysis of target genes for biological processes revealed that the downregulated genes were highly concentrated in chemotaxis, locomotory behavior, and inflammatory response biological processes. B. KEGG pathway analysis of target genes showed that the downregulated genes are enriched in several pathways including the NOD-like receptor signaling pathway and chemokine signaling pathway.
