# Supplementary material for: Protective Effects of Oridonin on Acute Liver Injury via Impeding Posttranslational Modifications of Interleukin-1 Receptor-Associated Kinase 4 (IRAK4) in the Toll-Like Receptor 4 (TLR4) Signaling Pathway
Source: Mediators Inflamm. 2019 Sep 12;2019:7634761. doi: 10.1155/2019/7634761 (PMC6757283; doi:10.1155/2019/7634761)
Supplement: Supplementary Materials — See Supplementary Tables S1-S2 in the Supplementary Materials for comprehensive image analysis. Figure S1: A. GO analysis of target genes for biological processes revealed that the downregulated genes were highly concentrated in chemotaxis, locomotory behavior, and inflammatory response biological processes. B. KEGG pathway analysis of target genes showed that the downregulated genes are enriched in several pathways including the NOD-like receptor signaling pathway and chemokine signaling pathway. [file 7634761.f1.docx]

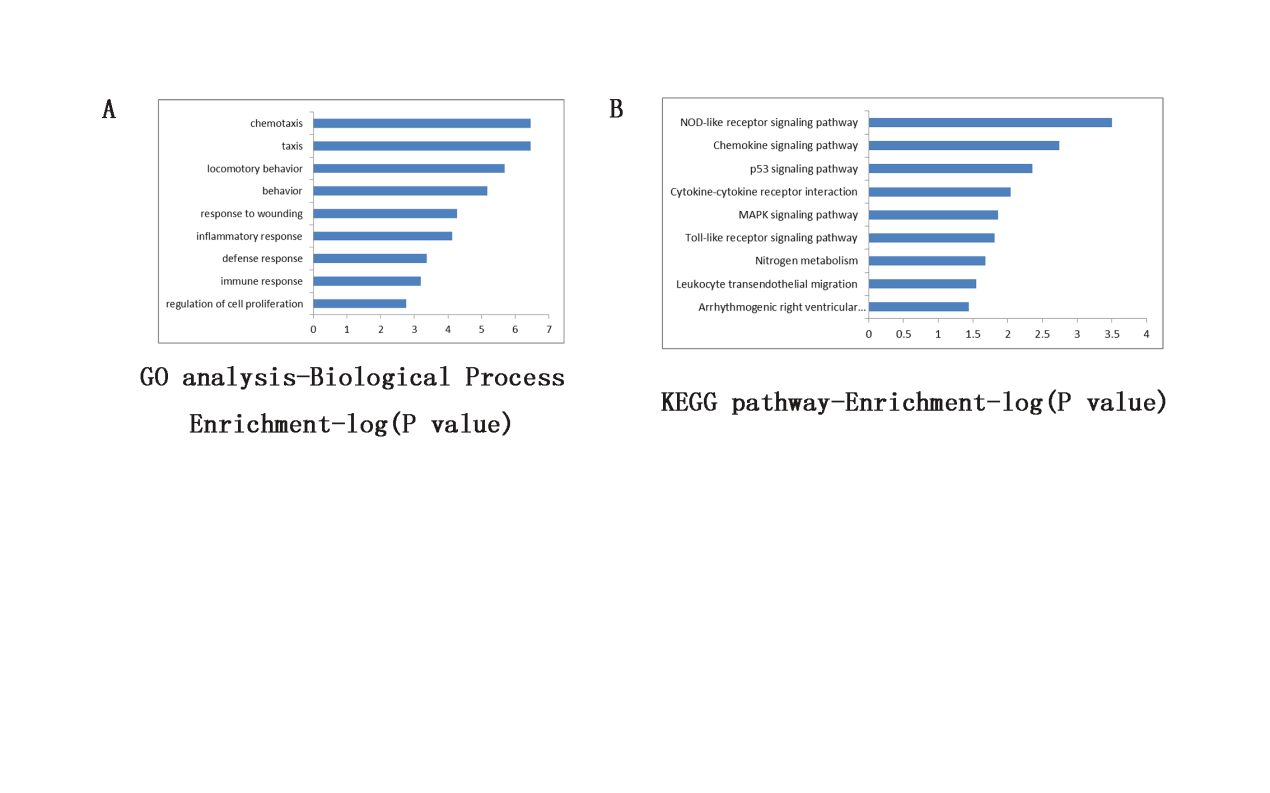


**Figure S1. A.** GO analysis of target genes for biological processes revealed that the down-regulated genes were highly concentrated in chemotaxis, locomotory behavior and inflammatory response biological processes. **B.** KEGG pathways analysis of target genes showed that the down-regulated genes enriched in several pathways including the NOD-like receptor signaling pathway and chemokine signaling pathway.
